# Different Intensity Exercise Preconditions Affect Cardiac Function of Exhausted Rats through Regulating TXNIP/TRX/NF-ĸB_p65_/NLRP3 Inflammatory Pathways

**DOI:** 10.1155/2020/5809298

**Published:** 2020-06-08

**Authors:** Yuemin Li, Peng Xu, Yang Wang, Junshi Zhang, Mei Yang, Yumei Chang, Ping Zheng, Heling Huang, Xuebin Cao

**Affiliations:** ^1^Department of Cardiology, The Hospital of the 82nd Group Army, Baoding 071000, Hebei, China; ^2^Department of Clinical Pharmacy, The Hospital of the 82nd Group Army, Baoding 071000, Hebei, China; ^3^Department of Central Laboratory, The Hospital of the 82nd Group Army, Baoding 071000, Hebei, China; ^4^Department of Gynaecology, The Hospital of the 82nd Group Army, Baoding 071000, Hebei, China

## Abstract

**Objective:**

To investigate whether exercise preconditioning (EP) improves the rat cardiac dysfunction induced by exhaustive exercise (EE) through regulating NOD-like receptor protein 3 (NLRP3) inflammatory pathways and to confirm which intensity of EP is better.

**Method:**

Ninety healthy male Sprague Dawley rats were randomly divided into five groups: a control group (CON), exhaustive exercise group (EE), low-, middle-, and high-intensity exercise precondition and exhaustive exercise group (LEP + EE, MEP + EE, HEP + EE group). We established the experimental model by referring to Bedford's motion load standard to complete the experiment. Then, the pathological changes of the myocardium were observed under a light microscope. Biomarker of myocardial injury in serum and oxidative stress factor in myocardial tissue were evaluated by ELISAs. The cardiac function parameters were detected using a Millar pressure and volume catheter. The levels of thioredoxin-interacting protein (TXNIP), thioredoxin protein (TRX), nuclear transcription factor kappa B_p65_ (NF-ĸB_p65_), NLRP3, and cysteinaspartate specific proteinase 1 (Caspase-1) protein in rats' myocardium were detected by western blotting.

**Results:**

1. The myocardial structures of three EP + EE groups were all improved compared with EE groups. 2. The levels of the creatine phosphating-enzyme MB (CK-MB), reactive oxygen species (ROS), interleukin-6 (IL-6), C-reactive protein (CRP), and tumor necrosis factor alpha (TNF-*α*) in three EP + EE groups were all increased compared with CON but decreased compared with the EE group (*P* < 0.05). 3. Compared with the CON group, slope of end-systolic pressure volume relationship (ESPVR), ejection fraction (EF), and peak rate of the increase in pressure (d*P*/d*t*_max_) all dropped to the lowest level in the EE group (*P* < 0.05), while the values of cardiac output (CO), stroke volume (SV), end-systolic volume (Ves), end-diastolic volume (Ved), and relaxation time constant (Tau) increased in the EE group (*P* < 0.05). 4. Compared with the CON group, the expression levels of TXNIP, NF-ĸB_p65_, NLRP3, and Caspase-1 all increased obviously in the other groups (*P* < 0.05); meanwhile, they were all decreased in three EP + EE groups compared with the EE group (*P* < 0.05). 5. NLRP3 was positively correlated with heart rate, IL-6, and ROS, but negatively correlated with EF (*P* < 0.01).

**Conclusion:**

EP protects the heart from EE-induced injury through downregulating TXNIP/TRX/NF-ĸB_p65_/NLRP3 inflammatory signaling pathways. Moderate intensity EP has the best protective effect.

## 1. Introduction

Exhaustive exercise (EE) refers to the continuous exercise in the overload state of the body. People who often engage in high-intensity exercise, such as athletes and soldiers, will be going to be EE. EE can cause a series of adverse reactions, including oxidative stress and myocardial inflammation [1]. It also can lead to oxidative stress and increase the production of reactive oxygen species (ROS) [2]. ROS is important for cellular homeostasis, in that it intervenes as cell signaling body in diverse pathways [3]. ROS raises thioredoxin interactions protein (thioredoxin-interacting protein, TXNIP) [4]. As a negative regulator of the thioredoxin protein (TRX) antioxidant system, TXNIP interacts with TRX in dormant cells and keeps it in an inactive state to maintain the balance of oxidation [5, 6]. Reactive oxygen species-thioredoxin-interacting protein (ROS-TXNIP) can form ROS-TXNIP inflammatory body axis, which can activate a nuclear transcription factor kappa B_p65_ (NF-ĸB_p65_) and regulate inflammation [7, 8]. NF-ĸB_p65_ is a powerful proinflammatory transcription factor that can trigger the activation of intracellular signal transduction [9]. The activated NF-ĸB_p65_ combines with the two binding sites of NOD-like receptor protein 3 (NLRP3) promoter region and activates the NLRP3 inflammatory corpuscle [10]. The activation of NLRP3 inflammasome accelerates the release of cysteinaspartate specific proteinase 1 (Caspase-1) and promotes the increase of downstream inflammatory factors [11]. Another study has reported that the inhibition of NLRP3-inflammasome could decrease the incidence of myocardial infarction and improve myocardial function in animal myocardial infarction models [12]. EE-induced inflammatory mediators can induce myocardial cell apoptosis and cardiac dysfunction [13, 14]. The inflammatory cytokines including tumor necrosis factor alpha (TNF-*α*), interleukin-6 (IL-6), interleukin 1 beta (IL-1*β*), and c-reactive protein (CRP) could directly or indirectly induce left ventricular dysfunction [15].

While exercise precondition (EP) can reduce the cardiac injury through tolerance training, it is one of the interventions to improve cardiac function [16–18]. Meng et al. have confirmed that EP could reduce the apoptosis of cardiac cells by inhibiting the TNF-*α* mediated apoptosis signaling pathway, and long-term EP had better effects than short-term EP. The EP anti-inflammatory strategy may be effective in improving cardiac dysfunction and heart failure after myocardial infarction [19]. Jiao et al. have proved that EP could regulate NLRP3 inflammation, reduce downstream inflammatory factors such as IL-1*β* and CRP, and play a protective role on the heart.

However, the effect of EE on the TXNIP/TRX/NF-ĸB_p65_/NLRP3 signaling pathway and the association of the inflammatory signaling pathway activated by EP and its cardiac protecting resist to the injury of EE all remain unclear. We hypothesize that EP can protect the heart from the cardiac injury induced by EE, and the protection is possibly triggered by the TXNIP/TRX/NF-ĸB_p65_/NLRP3 signaling pathway.

Meanwhile, there is also a controversy on which level of EP has a better protective effect on cardiac function [20]. It is still unclear which intensity or type of EP has the most significant protective effect on heart [21]. Therefore, we aimed to solve the above problem in this experiment.

## 2. Materials and Methods

### 2.1. Drugs and Main Instruments

The main reagents used in the present study are listed below. ROS kits were provided by Nanjings Mr Ng biological technology Co., Ltd. The CK-MB kit was provided by Wuhan huamei biological engineering Co., Ltd. IL-6, CRP, and TNF-*α* kits were purchased from the Wuhan optimal, born trade Co., Ltd. TXNIP, TRX, NF-ĸ B_p65_, NLRP3, and Caspase-1 antibody were obtained from Abcam Trading (Shanghai) Company Ltd. *β*-Actin antibody was supplied by Beijing zhongshan jinqiao biotechnology Co., Ltd.

The following main instruments were used in the present study: a PowerLab signal acquisition and analysis system, a MultiscanGO enzyme standard instrument (Thermo, United States), a Sigma 3k15 high-speed refrigerated centrifuge (SIGMA, Germany), a pressure volume catheter (SPR-838, Millar company, USA), a PowerLab data acquisition and analysis system (AD Instruments, Australia), vertical electrophoresis system (BIO-TEK, USA), transfer electrophoresis system (BIO-TEK, USA), a gel imaging system (Bio Spectrum), an image analysis system (Image-Pro Plus 4.1), a PowerLab data acquisition and analysis system (AD Instruments, Australia), a bioelectric amplifier (AD Instruments, Australia), and a needle electrode (AD Instruments, Australia).

### 2.2. Establishment and Grouping of Animal Models

Ninety male Sprague Dawley rats (200 ± 30 g) were provided by the Academy of Military Medical Sciences; License number: SCXK (Beijing)-2012-004. All experiments were conducted in compliance with the guide for the Care and Use of Laboratory Animals and approved by the Ethics Committee for the Use of Experimental Animals at the Hospital of the 82nd Group Army. Standard rodent dry feed was provided ad libitum, the indoor temperature was maintained at 18 to 22°C, and the relative humidity was maintained at 40 to 55%. SD rats were adaptive fed for 1 week and then randomly divided into 5 groups (*n* = 18): the control group (CON), exhaustive exercise group (EE), low-intensity exercise preconditioning + exhaustive exercise group (LEP + EE), middle-intensity exercise preconditioning + exhaustive exercise group (MEP + EE), and intense exercise preconditioning + exhaustive exercise group (HEP + EE group), following the standards of Bedford exercise load to establish the animal sport models [22]. In the LEP + EE group, the run slope was 3° and the speed was 30 m/min, equivalent to 40%∼50% VO_2max_. In the MEP + EE group, the run slope was risen to 6° and speed was risen to 32 m/min, equivalent to 65%∼75% VO_2max_. In the HEP + EE group, the run slope was also 6° and the speed was 36 m/min, equivalent to 90%∼95% VO_2max_. Exercise was followed for 8 weeks. Then, the EE, LEP + EE, MEP + EE, and HEP + EE groups run with the slope of 6° and the speed of 36 m/min until exhaustion. Criteria for judging exhaustion: the rats were left behind the runway more than 10 times in a row, and the photoelectric acoustic stimulation was invalid. Ten of the 18 animals in each group were used for the pressure volume catheter detection of cardiac function, which was an invasive experiment. These animals were euthanized after the experiment. Serum, electrocardiogram and myocardial specimens were collected from the remaining animals (*n* = 8 animals per group). There are two mice lost, respectively, in the EE group, LEP + EE group, and MEP + EE group. Also, the CON group and HEP + EE group have no lost mice (CON, HEP + EE *n* = 10; EE, LEP + EE, MEP + EE *n* = 8).

### 2.3. Collection and Preparation of Serum and Myocardial Samples

Rats were subjected to abdominal anesthesia with pentobarbital sodium (40 mg/kg), the chest was opened, and the blood was collected from the inferior thoracic vena cava. The blood was centrifuged at 3000 r/min for 20 minutes; the supernatant was collected and stored in a −80°C freezer until the detection of serum indicators.

Then, the hearts were quickly removed and washed with cold saline. Tissues were stored individually in a refrigerator at −80°C until western blot detection.

### 2.4. The Structure of the Myocardium Was Observed by Using an Optical Microscope

All rats were anesthetized with pentobarbital sodium (40 mg/kg, intraperitoneal injection). The hearts were quickly removed and washed with cold saline. Myocardial tissue of the left ventricle was collected for light microscopy analysis. The tissue was fixed with 10% formaldehyde, paraffin-embedded, sectioned, dehydrated with different concentration gradients of alcohol, stained with HE, and observed by the light microscopy.

### 2.5. Enzyme-Linked Immunoassays for ROS, TNF-*α*, IL-6, CRP, and CK-MB in Rat Serum

The serum was removed from the −80°C freezer and thawed. Enzyme-linked immunosorbent assays were performed according to the instructions included in the kits. The OD value of each sample was measured at 450 nm. The OD value for the standard was measured, and a standard curve was constructed with the OD value on the *y*-axis and the concentration on the *x*-axis. The concentration of the indicated marker in each sample was obtained from the standard curve.

### 2.6. Determination of Cardiac Function Parameters with a Pressure Volume Catheter

Rats were anesthetized with pentobarbital sodium (40 mg·kg^−1^, i.p.), and the closed-chest approach was chosen for catheter insertion [23]. The animal was fixed in the supine position on the operating table. The skin of the neck was disinfected prior to a midline neck incision, and the trachea was separated and intubated. The right carotid artery was separated from the common carotid artery. Two 4-0 silk threads were sewn through the common carotid artery, and one of the silk threads was used to ligate the proximal end of the carotid artery. A cut was made at the end of the heart to complete the knot. The pressure volume catheter was inserted through the incision into the left chamber along the inverse blood flow of the carotid artery and calibrated with MPVS control software. The left ventricular pressure volume waveform of the anesthetized rats was recorded with Chart 7 software in real-time. Vessels and catheters were fixed with another silk thread. The baseline data was recorded for 15 minutes. The abdominal skin was disinfected, a median incision was made, the inferior vena cava was occluded, and changes in the waveform were recorded. A 20 *μ*l solution of 30% NaCl was rapidly injected into the anterior jugular vein, and pressure-volume waveform changes were recorded. The first 4 holes of a calibration cuvette with known diameters (provided by the manufacturer) were quickly filled, and the catheter tip was submerged in fresh heparinized warm blood. The conductance changes in the volume channel were recorded, and the volume was then calculated.

The cardiac output (CO), heart rate (HR), end-systolic pressure (Pes), end-systolic volume (Ves), end-diastolic volume (Ved), stroke volume (SV), left ventricular development pressure (Pdev), ejection fraction (EF), peak rate of pressure rise (d*P*/d*t*_max_), peak rate of pressure decline (−d*P*/d*t*_min_), slope of the end-systolic pressure volume relationship (ESPVR), and relaxation time constant (Tau) were detected. The pressure volume loop (PV Loop) was drawn with pressure on the *Y*-axis and volume on the *X*-axis.

### 2.7. Western Blot Analysis of TXNIP, TRX, NF-*κ*B_p65_, NLRP3, and Caspase-1 Levels in the Left Ventricular Myocardium

The heart was removed from the −80°C freezer, and the left ventricular myocardial tissue was sheared on ice, minced with fine scissors, and 50 mg was removed and mixed with lysis buffer containing protease inhibitors and a phosphatase inhibitor. The solution was intermittently homogenized with an electric homogenate machine for 1 minute, incubated on ice for 30 minutes, and centrifuged at 12,000 r/min for 20 minutes at 4°C. The supernatant was then placed in a 0.5 ml centrifuge tube. The nucleoproteins were extracted according to the manufacturer's instructions. The protein concentrations were determined by the bicinchoninic acid (BCA) method with bovine serum albumin used as the standard. Then, the protein samples were diluted to the same volume and heated at 100°C for 5 minutes after the addition of an equal volume of loading buffer. The denatured protein samples were separated by SDS/polyacrylamide gel electrophoresis (SDS-PAGE) at 100 V for 2 h and transferred to polyvinylidene fluoride (PVDF) membranes. Membranes were blocked with blocking buffer containing 5% skim milk at room temperature for 1 hour and then incubated with primary antibodies overnight at 4°C. After the membranes were washed with Tris-buffered saline (TBS) containing Tween three times, they were incubated with secondary goat anti-mouse IgG antibodies conjugated to horseradish peroxidase for 1 h at room temperature and then exposed to enhanced chemiluminescence (ECL) for 1 to 2 min to detect the bands. A gel imaging system was used to capture images for the quantitative analysis, and grayscale values were determined.

### 2.8. Statistical Analysis

The data are presented as means ± SD. SPSS20.0 statistical software was used to analyze all experimental data. Single factor analysis of variance was used for comparisons of multiple means after a one-way ANOVA and a homogeneity test were first performed. Comparisons of mean values between two groups were performed using the LSD test if the variance was equal or Dunnett's T3 method if the variance was unequal. Correlation analysis was performed by calculating Pearson's correlation coefficients. Also, the single factor regression analysis was performed. *P* < 0.05 was considered to indicate a significant difference in all statistical methods.

## 3. Results

### 3.1. The Microstructure of Myocardial Tissue in Rats with Different Intensity EP

**Figure 1:**
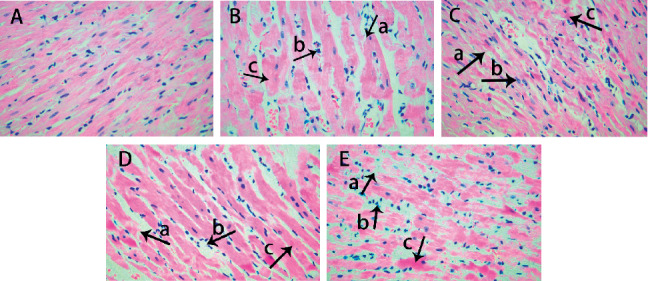
Microstructure of the myocardium using an optical microscope in each group of rats (HE ×400). (a) The control group, (b) the exhaustive exercise group, (c) the low-intensity exercise preconditioning + exhaustive exercise group, (d) the middle-intensity exercise preconditioning + exhaustive exercise group, and (e) the intense exercise preconditioning + exhaustive exercise group. n = 8 per group. (A) The arrow shows myocardial rupture. (B) The arrow shows myocardial cell edema and inflammatory infiltration. (C) The arrow shows myocardial hyperplasia and edema.

In the Con group, myocardial fibers arranged neatly and the cardiomyocyte membranes showed integrity. The interstitial was not edema, muscle membrane was not damaged, and there was no myocardial cell swelling or inflammatory cell infiltration (Figure 1(a)). EE Group: the myocardial staining was not uniform obviously, and a large number of myocardial fiber structures were broken and multiple myocardial fibers were disordered (Figure 1(b) A). Also, a large number of myocardial cells had edema and necrosis and were infiltrated by inflammatory cell (Figure 1(b) B). Meanwhile, mesenchymal fibers had moderate hyperplasia and edema (Figure 1(b) C). The results of LEP + EE, MEP + EE, and HEP + EE groups were all better than those of EE groups (Figures 1(c)–1(e)), and the improvement in the MEP + EE group was the most significant (Figure 1(c)).

### 3.2. The Effect of Different Intensity EP on Serum ROS, TNF-*α*, IL-6, CRP, and CK-MB Levels of Exhausted Rats

**Figure 2:**
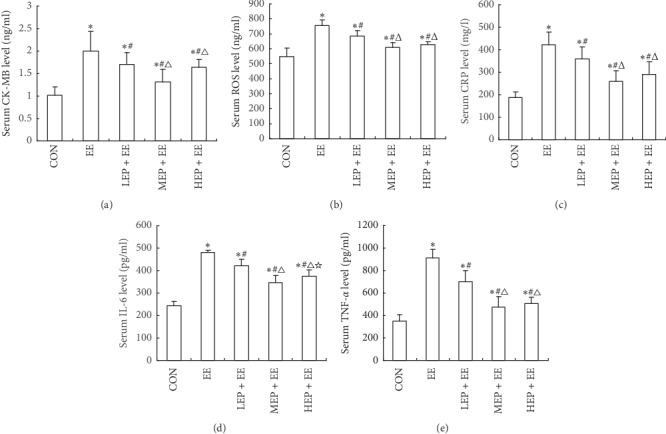
The effect of EP with different intensities on the serum (a) CK-MB, (b) ROS, (c) CRP, (d) IL-6, and (e) TNF-*α* levels in exhausted rats.

Compared with the CON group, the serum levels of ROS, TNF-*α*, IL-6, CRP, and CK-MB in the EE, LEP + EE, MEP + EE, and HEP + EE groups were all increased with statistical significance (*P* < 0.05). Compared with the EE group, the contents of ROS, TNF-*α*, IL-6, CRP, and CK-MB in the LEP + EE, MEP + EE, and HEP + EE groups were all decreased significantly (*P* < 0.05). Compared with LEP + EE group, the ROS, TNF-*α*, IL-6, and CRP in MEP + EE and HEP + EE groups all decreased prominently (*P* < 0.05). Compared with MEP + EE group, the CK-MB and IL-6 significantly increased in HEP + EE groups (*P* < 0.05) (Figure 2).

The data are presented as means ± SD, *n* = 8 per group. ROS: reactive oxygen species; TNF-*α*: tumor necrosis factor alpha; IL-6: interleukin-6; CRP: C-reactive protein; CK-MB: creatine phosphating-enzyme MB. CON: the control group, EE: the exhaustive exercise group, LEP + EE: the low-intensity exercise preconditioning + exhaustive exercise group, MEP + EE: the middle-intensity exercise preconditioning + exhaustive exercise group, and HEP + EE: the group intense exercise preconditioning + exhaustive exercise group. ^*∗*^*P* < 0.05, compared with the CON group; ^#^*P* < 0.05, compared with the EE group; ^Δ^*P* < 0.05, compared with the LEP + EE group; ^☆^*P* < 0.05, compared with the MEP + EE group.

### 3.3. The Effect of Different Intensity EP on the Cardiac Function Parameters of Exhausted Rats

Compared with the CON group, ESPVR, EF, and d*P*/d*t*_max_ all dropped to the lowest level in the EE group with statistically significant difference (*P* < 0.05), while the levels of CO, SV, Ves, Ved, and Tau increased significantly (*P* < 0.05). SV, Ves, Ved, and ESPVR in the LEP + EE, MEP + EE, and HEP + EE groups were all significantly different from those in the EE group (*P* < 0.05), and CO in the MEP + EE group was obviously lower than that in the HEP + EE group (*P* < 0.05). CO, SV, Ves, Ved, and Tau of the MEP + EE group were all the lowest among EP groups (Table 1).

### 3.4. The Effect of EP on the Expression of TXNIP, TRX, NF-*κ*Bp65, NLRP3, and Caspase-1 in the Myocardium

**Figure 3:**
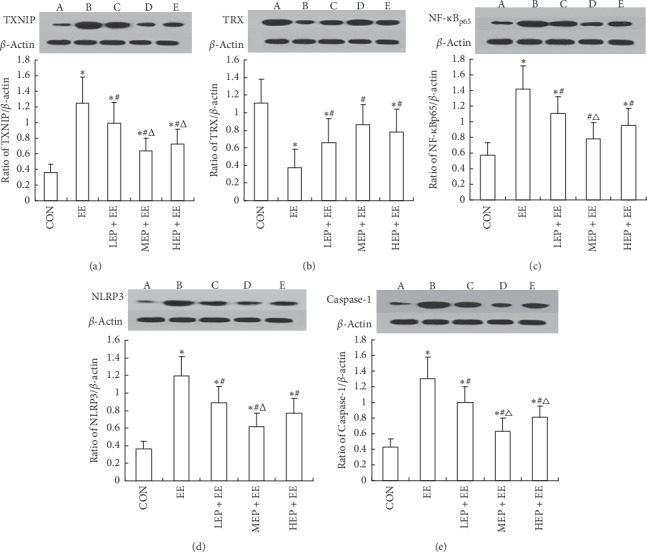
The comparisons with the relative expressions of myocardial protein TXNIP, TRX, NF-ĸB_p65_, NLRP3, and Caspase-1 in each group rats (*n* = 8, $\bar{x}\pm s$). (a) Ratio of TXNIP/*β*-actin in rats' myocardium; (b) ratio of TRX/*β*-actin in rats' myocardium; (c) ratio of NF-ĸB_p65_/*β*-actin in rats' myocardium; (d) ratio of NLRP3/*β*-actin in rats' myocardium; and (e) ratio of Caspase-1/*β*-actin in rats' myocardium. *β*-Actin: protein internal reference; TXNIP: thioredoxin-interacting protein; TRX: thioredoxin protein; NF-ĸB_p65:_ nuclear transcription factor kappa B_p65_; NLRP3; NLRP3 inflammatory; and Caspase-1: cysteinaspartate specific proteinase 1. A: the control group, B: the exhaustive exercise group, C: the low-intensity exercise preconditioning + exhaustive exercise group, D: the middle-intensity exercise preconditioning + exhaustive exercise group, and E: the group intense exercise preconditioning + exhaustive exercise group.^*∗*^*P* < 0.05, compared with the CON group; ^#^*P* < 0.05, compared with the EE group; ^Δ^*P* < 0.05, compared with the LEP + EE group.

Compared with CON group, the TXNIP, NF-ĸB_p65_, NLRP3, and Caspase-1 all increased obviously, but TRX reduced significantly in EE, LEP + EE, and HEP + EE groups (*P* < 0.05). The expression level of TXNIP, NF-ĸB_p65_, NLRP3, and Caspase-1 in LEP + EE, MEP + EE, and HEP + EE groups was all higher than that of the EE group, while the level of TRX was lower than that of the EE group (*P* < 0.05). Compared with the LEP + EE group, the TXNIP and Caspase-1 increased in MEP + EE and HEP + EE groups (*P* < 0.05). In addition, the level of NF-ĸB_p65_ and NLRP3 in the MEP + EE group was lower than that of LEP + EE group (*P* < 0.05, Figure 3).

### 3.5. Analysis of the Correlations between ROS, Myocardial Protein NLRP3, Inflammatory Factors, and Cardiac Function Parameters in Rats with Different Intensity EP

By Pearson linear correlation analysis, NLRP3 was positively correlated with ROS in the CON group (*r* = 0.87, *P* < 0.01). In the MEP + EE group, NLRP3 was positively correlated with HR, (*r* = 0.75, *P* < 0.01) and negatively correlated with EF, (*r* = −0.89, *P* < 0.01). In the HEP + EE group, NLRP3 was positively correlated with IL-6, (*r* = 0.87, *P* < 0.01) and negatively correlated with EF, (*r* = −0.84, *P* < 0.01) (Table 2).

## 4. Discussion

In this experiment, we studied the effects of different intensity EP on improving the structure of myocardium, reducing myocardial injury, and improving cardiac function in the exhausted rats. We found that the mechanism was EP could inhibit oxidative stress and regulate TXNIP/TRX/NF-ĸB_p65_/NLRP3 inflammatory pathways to improve inflammatory state. Furthermore, the moderate intensity EP has the best effect on cardiac protection.

Sports medicine studies have shown that certain decompensated changes occurred in myocardial morphological structure and functional metabolism after repeated high-intensity exercise training. The rupture of myocardial fibers caused the release of CK-MB from cardiomyocyte to peripheral blood. Studies have shown that the serum level of CK-MB increased significantly after exhaustive swimming. Wang has confirmed that the content of CK-MB could reflect the degree of cardiac injury [24]. In this study, EP improved the severe damage and inflammatory infiltration in myocardial fiber structure and decreased the serum level of CK-MB in exhausted rats.

The increased ventricular wall pressure which is led by exhaustion can result in fatigue, and cardiac remodeling may occur [25, 26]. Under the overload pressure, the left ventricular diastolic function and contractile function were impaired in rats. The d*P*/d*t*_max_ dropped to the lowest level in the EE group. The level of CO is one of the key factors that determine the ability of exercise [27]. In case of impaired cardiac function, the body can maintain a relatively normal level of cardiac function in a short term through the Frank–Starling compensation mechanism [28]. The results of our experiment showed that CO, SV, Ves, and Ved all increased in exhausted rats. The increase of SV after strenuous exercise added cardiac pump blood volume to meet the metabolic needs of all important organs in the whole body, and it was consistent with previous research conclusions. ESPVR and EF were significantly decreased, which indicated that myocardial systolic function decreased. In addition, the experimental results were consistent with previous results that the parameter Tau value was a representative indicator of the left ventricular diastolic function, and it was inversely proportional to the active diastolic function of the left ventricle. Our results indicated that EP had a protective effect on ventricular systolic and diastolic functions, and the effect of moderate intensity EP was better.

In recent years, oxidative stress and the activation of inflammatory pathways have been considered to play important roles in the transformation of cardiac remodeling and heart failure [29]. Murry and colleagues found that EP could substantially reduce infarct size. They also reported that many authors reproduced the infarct-sparing effect of EP in several mammalians after this original observation in dogs, such as rat, mice, rabbit, swine, and goat. Furthermore, it has been demonstrated that EP could protect the heart against the damage caused by ischemia-reperfusion and improve vascular and coronary reactivity [30]. Recent studies have attempted to reduce the damage of cardiovascular disease by eliminating reactive oxygen species or modulating inflammation [31]. The inhibiting of oxidative stress and ROS plays a cardioprotective role [5]. At a molecular level, exercise modulates the NF-ĸB signaling axis and contributes to prevent cardiac hypertrophy and right ventricle diastolic dysfunction. EP provides a cardio protection through a shift of the NF-ĸB signaling. In addition, proinflammatory cytokines such as TNF-*α* may depress cardiac contractility by promoting hypertrophy, apoptosis, and fibrosis [6]. EP can alleviate patcardiac remodeling and cardiac dysfunction through inhibiting the activation of NF-ĸB_p65_ pathways [9, 32]. Our study showed that EP could protect cardiac function through reducing the expression of TXNIP, NLRP3, NF-ĸB_p65_, caspase-1, and the downstream inflammatory cytokines such as TNF-*α*, IL-6, and CRP.

EE causes damage to cardiac function [33]. Feriani et al. found significant improvement in inflammatory parameters and cardiac function indicators in exhausted rats after aerobic exercise training. In order to control the harmful effects of inflammation on cardiac function, we need to pay more attention to the regulation of inflammatory pathways and strictly control inflammatory pathways and their activators [34, 35]. This experiment showed that ROS was positively correlated with NLRP3, that was to say, oxidative stress was related to the inflammatory pathway of NLRP3. NLRP3 was positively correlated with IL-6, and this suggested that NLRP3 inflammation activation could increase the release of downstream inflammatory factors. NLRP3 was positively correlated with HR and negatively correlated with EF, and these indicated that NLRP3 affected cardiac function likely.

In summary, EP can be used as a prevention and treatment for improving the exercise-induced heart injury. EP with different intensity, especially the moderate intensity EP, had a better effect on regulating inflammatory pathways and protecting cardiac function; these provided a new research theory for exercise physiology and exercise cardiology, but whether high-intensity EP has local damage to the myocardium and cardiac function still needs to be further studied. In addition, how to translate these experimental theoretical knowledge into clinical application may be an important field of translational medicine research in the future.

## Figures and Tables

**Table 1:** The effect of EP with different intensity on cardiac function parameters in exhausted rats ($\bar{x}\pm s$, CON and HEP + EE, *n* = 10; EE, LEP + EE, and MEP + EE, *n* = 8).

	CON	EE	LEP + EE	MEP + EE	HEP + EE
*Parameter*					
CO (ml/min)	31.33 ± 6.89	62.78 ± 9.60^*∗*^	44.78 ± 6.87^*∗*^^#^	38.09 ± 9.54^*∗*^^#^	61.01 ± 12.78^*∗*▲☆^
SV (*µ*L)	96.69 ± 15.73	188.34 ± 39.33^*∗*^	105.97 ± 25.16^#^	99.17 ± 19.96^#^	117.17 ± 17.43^#^
Ves (*µ*L)	152.29 ± 23.86	235.97 ± 65.06^*∗*^	147.59 ± 69.20^#^	107.79 ± 35.70^#^	152.23 ± 28.17^#^
Ved (*μ*L)	220.48 ± 31.69	430.26 ± 88.97^*∗*^	236.35 ± 65.06^#^	226.61 ± 32.06^#^	262.66 ± 35.37^#^
Pes (mmHg)	111.79 ± 18.47	115.43 ± 27.70	109.72 ± 21.43	137.79 ± 18.62^*∗*^^#▲^	133.38 ± 20.69^*∗*▲^
Pdev (mmHg)	111.03 ± 18.73	96.44 ± 20.59	111.99 ± 19.13	139.81 ± 20.24^*∗*^^#▲^	135.41 ± 16.53^*∗*^^#▲^
HR (bpm)	320.41 ± 28.94	298.28 ± 58.59	344.33 ± 83.40	396.24 ± 12.65^*∗*^^#▲^	397.67 ± 23.54^*∗*^^#▲^

*Systolic indices*					
EF (%)	44.24 ± 8.80	40.07 ± 8.12^*∗*^	56.74 ± 15.98^*∗*^^#^	61.25 ± 8.74^*∗*^^#^	58.15 ± 7.33^*∗*^^#^
dP/dt_max_ (mmHg/s)	11025 ± 4472	7894 ± 2803^*∗*^	9518 ± 3299	13935 ± 2696^#▲^	13171 ± 2350^#▲^
ESPVR	0.81 ± 0.39	0.28 ± 0.12^*∗*^	0.73 ± 0.31^#^	0.78 ± 0.29^#^	1.03 ± 0.22^#▲^

*Diastolic indices*					
−d*P*/d*t*_min_ (mmHg/s)	−10718 ± 5866	−8346 ± 3093	−8985 ± 3987	−14068 ± 3221^#▲^	−11589 ± 2889
Tau (ms)	4.76 ± 5.10	11.03 ± 2.46^*∗*^	11.47 ± 3.37^*∗*^	8.34 ± 1.07^*∗*^	9.71 ± 1.85^*∗*^

CON: the control group (*n* = 10), EE: the exhaustive exercise group (*n* = 8), LEP + EE: the low-intensity exercise Epreconditioning + exhaustive exercise group (*n* = 8), MEP + EE: the middle-intensity exercise preconditioning + exhaustive exercise group (*n* = 8), HEP + EE: the group intense exercise preconditioning + exhaustive exercise group (*n* = 10). CO: cardiac output; SV: stroke volume; Ves: end-systolic volume; Ved: end-diastolic volume; Pes: end-systolic pressure; Pdev: left ventricular development pressure; HR: heart rate; EF: ejection fraction; dP/dt_max_: peak rate of the increase in pressure; ESPVR: slope of end-systolic pressure volume relationship; –d*P*/d*t*_min_: peak rate of the decrease in pressure; Tau: relaxation time constant; ^*∗*^*P* < 0.05, compared with the CON group; ^#^*P* < 0.05, compared with the EE group; ^▲^*P* < 0.05, compared with the LEP + EE group; ^☆^*P* < 0.05, compared with the MEP + EE group.

**Table 2:** Pearson's correlation analysis of NLRP3, HR, EF, IL-6, and ROS in rats with preadaptive exercise of different intensity (*r*, *n* = 8).

Group	HR (bpm)	EF (%)	IL-6 (pg/ml)	ROS
CON	0.25	−0.02	0.14	0.87^*∗∗*^
EE	0.08	−0.12	0.46	0.11
LEP + EE	0.03	−0.32	0.66	0.24
MEP + EE	0.75^*∗*^	−0.89^*∗∗*^	0.39	0.08
HEP + EE	0.56	−0.84^*∗∗*^	0.87^*∗∗*^	0.03

The data show Pearson's correlation coefficient (*r*), *n* = 8 animals per group. NLRP3: NLRP3 inflammatory; HR: heart Rate; EF: ejection fraction; IL-6: interleukin-6; ROS: reactive oxygen species. For groups, see the footnote to Table 1. ^*∗*^*P* < 0.05, ^*∗∗*^*P* < 0.01.

## Data Availability

All datasets analyzed to support the findings of the current study are available from the corresponding author upon reasonable request.

## References

[1] Chen C., Ma X., Yang C. (2017). Hypoxia potentiates LPS-induced inflammatory response and increases cell death by promoting NLRP3 inflammasome activation in pancreatic *β* cells. *Biochemical & Biophysical Research Communications*.

[2] Camiletti-Moirón D., Aparicio V. A., Aranda P., Radak Z. (2013). Does exercise reduce brain oxidative stress? A systematic review. *Scandinavian Journal of Medicine & Science in Sports*.

[3] Nancy V.-M., Ángel M.-G., Eduardo Osiris M.-S. (2019). Antioxidant and adaptative response mediated by Nrf2 during physical exercise. *Antioxidants (Basel)*.

[4] Choe J.-Y., Kim S.-K. (2017). Quercetin and ascorbic acid suppress fructose-induced NLRP3 inflammasome activation by blocking intracellular shuttling of TXNIP in human macrophage cell lines. *Inflammation*.

[5] Powers S. K., Smuder A. J., Kavazis A. N., Quindry J. C. (2014). Mechanisms of exercise-induced cardioprotection. *Physiology*.

[6] Nogueira-Ferreira R., Moreira-Gonçalves D., Silva A. F. (2016). Exercise preconditioning prevents MCT-induced right ventricle remodeling through the regulation of TNF superfamily cytokines. *International Journal of Cardiology*.

[7] Bi Q., Hou J., Qi P. (2016). TXNIP/TRX/NF-*κ*B and MAPK/NF-*κ*B pathways involved in the cardiotoxicity induced by Venenum Bufonis in rats. *Scientific Reports*.

[8] Haack K. K. V., Zucker I. H. (2015). Central mechanisms for exercise training-induced reduction in sympatho-excitation in chronic heart failure. *Autonomic Neuroscience*.

[9] Van O. N., Van G. H., Verdonckt M. (2017). Caspase-1 engagement and TLR-induced c-FLIP expression suppress ASC/Caspase-8-dependent apoptosis by inflammasome sensors NLRP1b and NLRC4. *Cell Reports*.

[10] Yin Y., Chen F., Wang W. (2017). Resolvin D1 inhibits inflammatory response in STZ-induced diabetic retinopathy rats: possible involvement of NLRP3 inflammasome and NF-*κ*B signaling pathway. *Molecular Vision*.

[11] Meng M. (2017). Digitoflavone (DG) attenuates LPS-induced acute lung injury through reducing oxidative stress and inflammatory response dependent on the suppression of TXNIP/NLRP3 and NF-*κ*B. *Biomedicine & Pharmacotherapy*.

[12] Yue R.-C., Lu S.-Z., Luo Y. (2019). Calpain silencing alleviates myocardial ischemia-reperfusion injury through the NLRP3/ASC/Caspase-1 axis in mice. *Life Sciences*.

[13] Dra R., Gomes M. J., Rosa C. M. (2017). N-acetylcysteine influence on oxidative stress and cardiac remodeling in rats during transition from compensated left ventricular hypertrophy to heart failure. *Cellular Physiology & Biochemistry International Journal of Experimental Cellular Physiology Biochemistry & Pharmacology*.

[14] Gregersen I., Askevold E. T., Sagen E. L. (2017). Interleukin 27 is increased in carotid atherosclerosis and promotes NLRP3 inflammasome activation. *PLoS One*.

[15] Li H., Miao W., Ma J. (2016). Acute exercise-induced mitochondrial stress triggers an inflammatory response in the myocardium via NLRP3 inflammasome activation with mitophagy. *Oxidative Medicine and Cellular Longevity*.

[16] Macnicol J. L., Lindinger M. I., Pearson W. (2017). A time course evaluation of inflammatory and oxidative markers following high intensity exercise in horses: a pilot study. *Journal of Applied Physiology*.

[17] Du S.-Q., Wang X.-R., Zhu W. (2018). Acupuncture inhibits TXNIP-associated oxidative stress and inflammation to attenuate cognitive impairment in vascular dementia rats. *CNS Neuroscience & Therapeutics*.

[18] Bonadei I., Sciatti E., Vizzardi E. (2018). Effects of ivabradine on endothelial function, aortic properties and ventricular-arterial coupling in chronic systolic heart failure patients. *Cardiovascular Therapeutics*.

[19] Fujisue K., Sugamura K., Kurokawa H. (2017). Colchicine improves survival, left ventricular remodeling, and chronic cardiac function after acute myocardial infarction. *Circulation Journal Official Journal of the Japanese Circulation Society*.

[20] Paz G. A., Iglesiassoler E., Willardson J. M. (2017). Postexercise hypotension and heart rate variability responses subsequent to traditional, paired set, and superset training methods. *Journal of Strength & Conditioning Research*.

[21] Maral R., Hamid R., Fatemeh R. (2019). The greater effect of high-intensity interval training versus moderate-intensity continuous training on cardioprotection against ischemia-reperfusion injury through Klotho levels and attenuate of myocardial TRPC6 expression. *BMC Cardiovascular Disorders*.

[22] Bedford T. G., Tipton C. M., Wilson N. C., Oppliger R. A., Gisolfi C. V. (1979). Maximum oxygen consumption of rats and its changes with various experimental procedures. *Journal of Applied Physiology*.

[23] Pacher P., Nagayama T., Bátkai P., Kass D. A. (2008). Measurement of cardiac function using pressure-volume conductance catheter technique in mice and rats. *Nature Protocols*.

[24] Wang X. W., Cao X. B., Hou C. C. (2013). A clinical study of exhaustive cardiac injury in a combat zone. *Chinese Journal of Integrated Traditional Chinese and Western Medicine First Aid*.

[25] Frasier C. R., Moore R. L., Brown D. A. (2011). Exercise-induced cardiac preconditioning: how exercise protects your achy-breaky heart. *Journal of Applied Physiology*.

[26] Paulus W. J., Tschöpe C. (2013). A novel paradigm for heart failure with preserved ejection fraction. *Journal of the American College of Cardiology*.

[27] Laroche D., Joussain C., Espagnac C. (2013). Is it possible to individualize intensity of eccentric cycling exercise from perceived exertion on concentric test?. *Archives of Physical Medicine and Rehabilitation*.

[28] Crisafulli A. (2017). The impact of cardiovascular diseases on the cardiovascular regulation during exercise in humans: studies on the metaboreflex elicited by the post-exercise muscle ischemia method. *Current Cardiology Reviews*.

[29] Zhang Y., Huang J., Yang X. (2017). Altered Expression of TXNIP in the peripheral leukocytes of patients with coronary atherosclerotic heart disease. *Medicine*.

[30] Lamont K. T., Somers S., Lacerda L. (2011). Is red wine a SAFE sip away from cardioprotection? Mechanisms involved in resveratrol- and melatonin-induced cardioprotection. *Journal of Pineal Research*.

[31] Narasimhan M., Rajasekaran N. S. (2017). Cardiac Aging-benefits of exercise, Nrf2 activation and antioxidant signaling, exercise for cardiovascular disease prevention and treatment. *Advances in Experimental Medicine and Biology*.

[32] Xu T., Zhang B., Yang F. (2015). HSF1 and NF-*κ*B p65 participate in the process of exercise preconditioning attenuating pressure overload-induced pathological cardiac hypertrophy. *Biochemical and Biophysical Research Communications*.

[33] Smenes B. T., Bækkerud F. H., Slagsvold K. H. (2018). Acute exercise is not cardioprotective and may induce apoptotic signalling in heart surgery: a randomized controlled trial. *Interactive Cardiovascular & Thoracic Surgery*.

[34] Ellwanger K., Becker E., Kienes I. (2018). The NLR family pyrin domain containing 11 protein contributes to the regulation of inflammatory signalling. *Journal of Biological Chemistry*.

[35] La A. G., Rakhit D. J., Claessen G. (2017). Exercise and the right ventricle: a potential achilles’ heel. *Cardiovascular Research*.

